# Nanoclay Effect into the Biodegradation and Processability of Poly(lactic acid) Nanocomposites for Food Packaging

**DOI:** 10.3390/polym13162741

**Published:** 2021-08-16

**Authors:** Helena Oliver-Ortega, Victor Vandemoortele, Alba Bala, Fernando Julian, José Alberto Méndez, Francesc Xavier Espinach

**Affiliations:** 1 Group LEPAMAP-PRODIS, Department of Chemical Engineering, University of Girona, c. M. Aurèlia Capmany, 61, 17003 Girona, Spain; fernando.julian@udg.edu (F.J.); jalberto.mendez@udg.edu (J.A.M.); francisco.espinach@udg.edu (F.X.E.); 2 KU LEUVEN, Gebroeders de Smetstraat 1, 9000 Gent, Belgium; victorvandemoortele@outlook.com; 3 UNESCO Chair in Life Cycle and Climate Change ESCI-UPF, Universitat Pompeu Fabra, Passeig Pujades 1, 08003 Barcelona, Spain; alba.bala@esci.upf.edu

**Keywords:** nanocomposites, nanoclays, PLA, biodegradation, processability

## Abstract

One of the most promising expectations in the design of new materials for food packaging is focused on the development of biodegradable systems with improved barrier character. In this sense PLA reinforced with nanoclay is a potential alternative to the use of conventional oil-derivative polymers due to the synergetic effect of the biodegradable character of PLA and the barrier-induced effect derived from the dispersion of nanoparticles. In this work, composite materials based on PLA and reinforced with bentonite nanoparticles (up to 4% *w*/*w*) (NC) have been prepared to produce films with improved barrier character against water vapor transportation. Additionally, the biodegradable character of the composites depending on the crystallinity of the polymer and percentage of NC have been evaluated in the presence of an enzymatic active medium (proteinase K). Finally, a study of the capacity to film production of the composites has been performed to determine the viability of the proposals. The dispersion of the nanoparticles induced a tortuous pathway of water vapor crossing, reducing this diffusion by more than 22%. Moreover, the nanoclays materials were in all the cases acceptable for food packing in terms of migration. A migration lower than 1 mg/m^2^ was obtained in all the materials. Nonetheless, the presence of the nanoclays in decreased biodegradable capacity was observed. The time was enlarged to more than 15 days for the maximum content (4% *w*/*w*). On the other hand, the incorporation of NC does not avoid the processability of the material to obtain film-shaped processed materials.

## 1. Introduction

Some polymeric materials, depending on the chemical stability and the additives, are suitable to be in contact with foods, as it is clear from the European Regulation (EC) No 1935/2004. Such kind of use requires specific characteristics of the final pieces to allow the development of the technical tasks for which they have been designed (mechanical performance, thermal resistance, transparency, if needed, etc.) but also others derived from the necessity of food safety and quality, especially in the case of packaging for fresh food.

Taking into account these requirements, the market is full of polymer materials, considered as single constituents or together with others, conforming multicomponent products [1,2], including independent capacities to develop a common target. That is the case of polyolefins such as polyethylene, high (HDPE) and low density (LDPE), polypropylene, polycarbonate (PC) and polyesters such as polyethylene terephthalate (PET). These polymers, non-biodegradable and derived from petroleum, are directly affected by the new European directive 2019/904 about plastics of single-use [3].

In this sense, polymer science and technology are immersed, for several years, in a change of paradigm in the use of raw material focused on the use of alternative polymer matrices friendlier with the environment. Thus, there is a strong research effort to find alternative polymer matrices to those produced from petroleum. These petroleum-based matrices are massively used and are directly responsible for pollution, emission of greenhouse gasses (GHGS) and consequently the global climate change [4].

Biodegradable, bio-based, renewable, compostable and other, terms are increasing in interest due to their different specific capacities to be degraded by the environment, produced from natural resources, or capacity to be compostable to allow elimination from the environment once their working life has finished [5]. That is the case of polyesters, such as poly(butylene succinate) (PBS), poly(ethylene succinate) (PES), poly(glycolide) (PGA), poly(hydroxyalcanoates) (PHAs) and poly(lactic acid) and their copolyesters. The remarkable interest in this polymer matrices is derived from the fact that they have ester groups (-COOR-) in the main polymer chain that is susceptible to be degraded under specific conditions. This degradability is mainly based on the hydrolytic susceptibility of ester groups included in aliphatic polymer chains and has been widely studied since several decades ago for very different applications [6,7,8,9].

However, biodegradability is not the only special characteristic that could be interesting for being used as packaging for fresh food, but barrier capacity is also needed [10]. Fresh food, such as meat, has a very short packing time (several days) due to the loss of nutritional capacities as well as organoleptic ones, due to the oxidation of myohemoglobin to methaemoglobin (meat darkening) [11].

One of the strategies to improve the barrier character of materials to be used as packaging is the incorporation of third species to modifying the pathway of diffusion of undesirable gases through the packaging [12,13], following the mechanism explained by Nielsen in 1967 [14]. The incorporation of particles in the nanoscale produces a tortuosity in the diffusion of gases by their dispersion in the matrix. This obstruction to the transit of gases through the material is associated with a longer diffusion pathway that takes a longer time to cross the packaging film.

Therefore, the fabrication of composite systems based on the structure of polyesters with hydrolytic susceptibility and reinforced with nanodispersed systems to induce barrier character is one of the potential ways to develop useful materials to be used as packaging. PLA is the most often used biodegradable polymer matrix [15,16,17,18,19,20,21], also including its copolymers [22,23], reinforced with nanostructured particles. In this sense, the nanoparticles that have demonstrated the best behavior against the diffusion of gases through the polymer are layered silicates and their organomodifications, due to their capacity to be exfoliated to promote intercalation between the polymer chains. Montmorillonite, hectorite, saponite, bentonite, cloisite and other silicates [24,25,26,27] are the most often studied. Alexandre et al. [28] define intercalation as a multilayer structure with alternating layers of nanosized silicates and polymer matrix characterized by the dispersion of the clay in the continuous polymer. When this dispersion acquires its maximum extension, the composite system is considered exfoliated or delaminated. Thus, the characterization of the higher or lower exfoliation capacity can be monitored by XRD diffraction. Nanoclays are also considered as nucleation agents, so they can modify the microstructure of the polymer including other properties such as barrier character [29], where increasing percentages of crystalline phase of PLA decreased values of water vapor transmission rate as expected. Naderi-Samani et al. [30] demonstrated that the improvement in the dispersion of 1–5% *w*/*w* of cloisite 20 A in polyamide–imide blends increased thermal stability attributed to the interaction of the nanoreinforcement with the matrix by H-bonding. Lendvai et al. [31] also corroborated that the incorporation of nanoclays to polymer matrices, starch in this case, also improved mechanical strength by adding bentonite in the range of 7–8% *w*/*w*, attributed to the increase of specific area of interaction reinforcement-matrix. An equivalent result was obtained by Sinha Ray et al. [22] but in terms of flexural strength, where the reinforcement of PLA with montmorillonite increased this property up to 26% by the incorporation of 4% *w*/*w* of the nanoreinforcement. A negative effect of the reinforcing effect is the loss of light transmission of the films due to the presence of the nanoparticles although can be modified by the application of thermal treatments such as quenching or isothermal crystallization [32] to improve it.

These nanocomposites have been found materials with improved barrier character against water vapor and oxygen transmission depending on the kind of nanoreinforcement added to the matrix [33,34]. In this sense, the best results have been obtained for composites based on starch reinforced with polar nanoclays [35] or even another kind of nanostructured reinforcement, such as nanofibrillated cellulose [36], where the incorporation of 0.36 wt% of this natural reinforcement to plasticized starch produced a decrease in water vapor transmission rate by 56% and 46% in the case of oxygen permeability. These properties of gas transmission also are influenced by the crystalline phase of the polymer, which can also be modified by the presence of the nanoclays as mentioned above.

The incorporation of nanoclays also produces a decrease in the biodegradable character of the polymer matrix that can be accelerated by the presence of enzymes. In the case of PLA composites, enzymes such as lipases [37], depolymerase [38,39] although the use of proteinase K is one of the most widespread [40,41,42,43]. The degradation catalyzed by such enzyme is focused mainly on the amorphous phase of the polymer, so during degradation, the crystalline phase of the polymer is increased [44].

Another huge field of study of materials for packaging is focused on the line of processing of the materials. A large amount of plastic products for food packaging is obtained by thermoforming [45]. Thermoforming involves heating a thermoplastic sheet or film to a temperature between its glass transition temperature and its melting temperature. Then, a mold with the shape of the product is put in contact with the film and a vacuum is applied to ensure full contact between the film and the mold. In the case of PLA, with glass transition, thermal crystallization and melting temperatures of 60, 100 and 160 °C respectively, special attention to changes in the structure of the film must be taken. The temperature must ensure enough molecular chain mobility but avoid crystallization [46,47]. Usually, thermoformed parts show heights half or equal to the shortest side, to ensure a proper forming and avoid tears due to critical loss of thickness. Another process used to obtain film is blown film extrusion. This process involves higher thickness loss than thermoforming and needs more material and more expensive equipment than thermoforming. This process is limited by the poor elongation capabilities of PLA which can cause the appearance of tears [48]. Thus, some efforts have been done to enhance the ability of PLA to deform, using plasticizers [49].

In this work, the authors analyzed the effect in the barrier, biodegradation and the processability properties of films produced from nanocomposites materials reinforced with a 2 and 4% *w*/*w* content of nanoclays. The nanoclays were introduced in the polymer matrix by the use of a masterbatch methodology instead of a direct melting mixture. It is known that directly mixed technologies presented disadvantages in comparison with soluble methodologies in terms of nanoclays dispersion and exfoliation. In addition, a previous study has shown the suitability of this methodology against direct melting to produce nanocomposites. Nonetheless, it is different from the production of films or thick samples. In addition, the food market is mainly interested in films for the barrier properties. Thus, it made interesting the study of such materials in a film product and the behavior in the biodegradation.

## 2. Materials and Methods

### 2.1. Materials

PLA used as a polymer matrix for the composite production was branch 3251D from NatureWorks (Naarden, the Netherlands). Nanoclays, in a powder form, were Bentonite with a particle size of 6 microns and a bulk density of 779 kg·m^−3^. The nanoclays were provided by Sigma Aldrich (Madrid, Spain). Proteinase K, the enzyme used for the biodegradation, Tris and chlorohydric acid used for the pH adjustment were also supplied by Sigma Aldrich.

### 2.2. Methodology

#### 2.2.1. Films Production

The composites materials were produced as published in previous work [20]. In summary, a masterbatch methodology was used to facilitate the production and dispersion of the nanoclays by melt mixing. The masterbatch materials were compounded with the nanoclay powder and the polymer in a concentration of 22% *w*/*w* using a Brabender plastograph (Duisburg, Germany). The compounding was carried out at 190 °C and low speed (45 rpm) for 5 min. Afterward, the masterbatch was diluted to 2 and 4% *w*/*w* nanocomposites in a Gelimat Kinetic mixer. The masterbatch and the PLA were loaded at low speed (300 rpm) and then increased to 2500 rpm. The obtained blend was discharged, pelletized and dried previous to the film production. The films were produced in hot press equipment (Fontijne Grotnes, Vlaardingen, the Netherlands). A quantity of 5 to 10 g, depending on the material, was placed between two metal plates and placed in the hot press at 190 °C. After that pre-heating process, the plates were pressed with a force of 60 kN for 1 min and at the same temperature. Then, while the pressure was kept, the temperature of the hot plates was decreased until room temperature to obtain the maximum amorphous content in the films. In the case of crystallized PLA, during the cooling down an additional step was performed. The films were initially cool down until 110 °C and this temperature was maintained for 40 min to allow the material to crystallize. Afterward, the film was cool down until room temperature. The films obtained had a thickness of around 0.1 mm.

#### 2.2.2. Water Vapor Transmission Rate (WVTR) and Global Migration Test

Films were characterized in terms of Water Vapor Transmission Rate (WVTR) and transmittance was measured to characterize their barrier properties. Films were placed in an apparatus with a circular exposed area of 38.5 cm^2^. In the bottom part, dried silica gel was placed and the apparatus was closed and placed in a climatic chamber at 23 °C and 50% RH. The test was performed by triplicate using different films for each studied sample. The gain in mass was measured at certain times and the WVTR was calculated from the slope of the relationship between the gain mass and the time as:

(1) $$\mathrm{WVTR}\;\left(g\cdot m^{-2}\cdot {\mathrm{day}}^{-1}\right)=\frac{24\;x}{A\;y}$$

where *x* is the mass gain, *y* is time in hours and *A* is the exposed area of the film.

European regulation (EU) 10/2011 for plastics in contact with food limits the global migration to 10 mg/dm^2^. Films samples of PLA and PLA + 4%NC were tested with water (as reference) and simulant B (acetic acid 3% *w*/*v*) at room temperature (20 °C) as indicated for refrigerated and fresh food with high direct contact. The exposure time was 10 days, indicated for long contacts (from 3 to 30 days). The test was carried out by triplicate.

#### 2.2.3. Biodegradation Test

A biodegradation test was performed using proteinase K as a biodegradation agent. A concentration of 200 mg·L^−1^ of enzyme in the buffer solution was used as observed in the literature [41,42,43]. The buffer was prepared with a concentration of 0.1 M of Tris in MiliQ water and adjusted to a pH 8.6 by the addition of some drops of diluted hydrochloric acid. The solution was prepared before its use to avoid enzyme degradation.

Samples of 15 × 15 × 0.1 mm^3^ of films, previously dried in a vacuum system at room temperature, weighted and placed in closed bottles and then, 10 mL of the enzyme solution was added. The samples were placed in an oven at 37 °C and removed at a certain time. For each time and material, a total of 3 films with adequate measures were prepared. The mass loss of the film samples was measured as:

(2) $$\mathrm{Mass}\;\mathrm{loss}\;\left(\%\right)=\frac{m_{t}-m_{0}}{m_{0}}\times 100$$

where *m*_0_ is the initial dried mass and *m_t_* is the dried mass of the sample after removing from the solution. Parallel, the pH of the solution was measured at the same time that the sample was removed.

#### 2.2.4. Structural and Thermal Characterization of the Films

The structural changes in the films were characterized using Transformed Infrared Spectroscopy (FT-IR). FT-IR data were collected in a Bruker Alpha FT-IR spectrometer (FT-IR) (Bruker, Madrid, Spain) using an attenuated total reflectance (ATR) cell. The analyzed range was from 400 to 4000 cm^−1^. Changes in the thermal behavior of PLA and PLA nanocomposites before and after the biodegradation process were checked by DSC. Tests were carried out in a Q2000 DSC from TA Instruments (New Castle, DE, USA) from 30 to 190 °C with a heating and cooling rate of 10 °C·min^−1^. Two heating cycles were performed and an inert atmosphere (N_2_) was used during the experiment. The crystallinity of the samples was obtained by using 93.6 J·g^−1^ as the enthalpy for a 100% crystalline PLA sample [50].

#### 2.2.5. Processability

The plug assist thermoforming of PLA sheets was made in Formtech equipment. One frame measuring 127 × 127 mm^2^ was used to fasten the films to the machine. The equipment has three heating areas and only the first, which coincides with the central area was used. The thermostat was put in the fifth position, allowing temperatures from 260 to 270 ºC. Heating the films took approximately 30 s for PLA films and 15 s for the composites. Once the film had the expected temperature two processability tests were made. On the one hand, a mold with a square base pyramid of 50 × 50 mm^2^ and a 50 mm height was used to evaluate the thermoformability of the films. On the other hand, a solid parallelepiped was pushed up from the vacuum chamber to assess the films of stand high deformation and simulate a blown film extrusion. The parallelepiped was centered concerning the film and has 170 × 66 × 75 mm^3^ measures. The 170 height ensures a maximum elongation of the film up to 151 mm. In both cases, once the final height was reached, the vacuum pump was started to finish the thermoforming process. The process took an average of 3 s. The experiment was designed to measure the capabilities of the films to endure high deformations. The experiment was performed by triplicate for each sample and height.

In the experiments the values that were registered were: the maximum height of node assist formability (HNAF), the first height at which a tear appears in the thermoformed film (DFT), the aerial draw ratio (ADR) and the film wall thickness reduction. All distance measures were made with a digital caliper and the thickness with a micrometer. HNAF measures the ability of a film to deform. The film is pushed by a parallelepiped node up to the height where the film breaks. The formation of tears in the film surface due to excessive stretching hinders the use of the materials for thermoforming or film extrusion blown molding. Once the HNAF experiment is finished, DFT measures the distance between the top of the formed shape and the first tear. The higher the distance the better will be the ability of the materials to deform without tears appearing.

## 3. Results and Discussion

### 3.1. Water Vapour Transmission Rate (WVTR) and Global Migration

**Figure 1:**
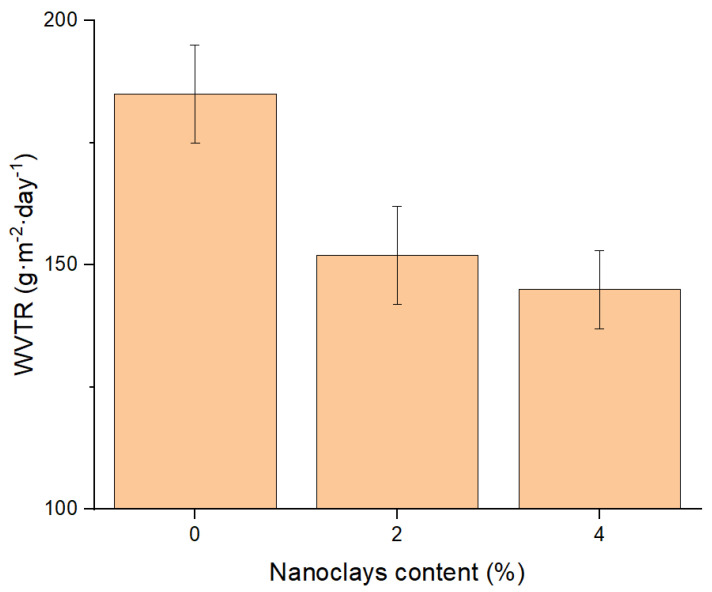
WVTR of PLA and its nanocomposites regarding the nanoclay contents and normalized to 25 µm.

WVTR of the PLA, PLA + 2%N and PLA + 4%N was measured and the results are shown in Figure 1. The thickness of the films was normalized to a value of 25 μm following the expression:

(3) $$\mathrm{WVTR}\;\left(g\cdot m^{-2}\cdot {\mathrm{day}}^{-1}\right)\;\mathrm{in}\;25\;\mu m\;\mathrm{films}=\mathrm{WVTR}\times \frac{l}{25}$$

where *l* is the thickness of the tested film.

The WVTR of the PLA films was reduced by the addition of the nanoclays. The results were expected as it has been previously reported that an adequate dispersion and intercalation of the nanoclays made a torturous pathway? for the gases. The addition of a 2% *w*/*w* of nanoclays represents a reduction of around 22% in comparison with the neat PLA in normalized films of 25 µm. However, the reduction has not the same trend in the case of 4% *w*/*w* reinforced nanocomposite, as the barrier properties against vapors were slightly improved from the 2 to the 4 *w*/*w*% reinforcement content. The results could be related to the higher agglomeration and lower intercalation effect in this nanocomposite, as it has been observed in previous work [20]. It is difficult to obtain an adequate intercalation of nanoclays by melt mixing methodologies. This difficulty is increased when the nanoclay content is raised in the composition. Additionally, the huge differences in the polarity of the matrix and the reinforcement limit its dispersion. The differences in the intercalation were observed previously in another work [20]. The improvement in the barrier properties is still far from other plastics used for packagings such as polyethylene (PE), which has a poor affinity for water which enhances that property, or polyethylenetherephtalate (PET) [51,52,53]. Nonetheless, the PLA materials are competitive against other bioplastics used like the thermoplastic starch or copolymers of polybutylene adipate terephthalate and other biobased polyesters [54]. Moreover, PLA, in general, shows a better performance than these plastics, thus, the use of nanoclays nanocomposites could reduce the material volume used, as thinner films could obtain the same performance.

A global migration test was carried out to check the suitability of these materials to be used in food packaging. The results obtained in mg/kg of aliment and mg/dm^2^ are shown in Table 1:

The results showed that all the samples reported results lower than 1 mg/kg and 1 mg/dm^2^. Although there are differences obtained, the PLA + 4%NC showed values slightly superiors to PLA, the values are far from the minimum required by the EU regulations. Thus, PLA nanocomposites could be used in food packaging for refrigerated products for long periods.

### 3.2. Biodegradation Test

**Figure 2:**
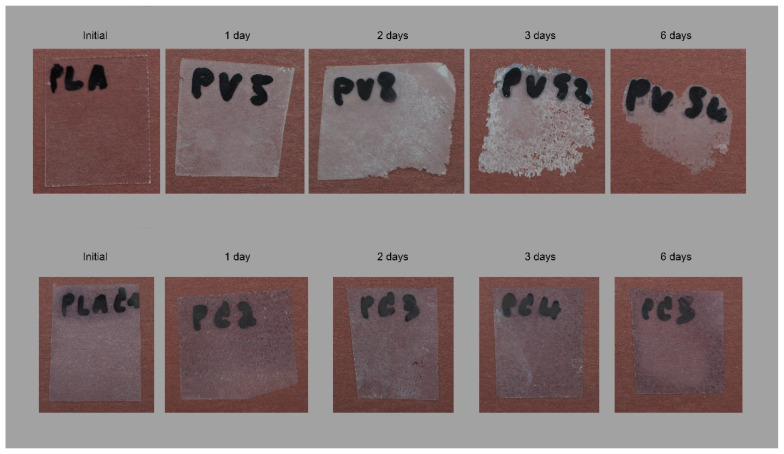
Optical analysis of the biodegradation test in amorphous PLA (**up**) and PLA crystallized (**down**).

The biodegradation test was initially carried out in neat PLA and crystalized PLA to assess the effect of the crystallinity on the degradation performance of proteinase K. Initially an optical analysis of the samples was done. Figure 2 shows the changes in the aspect of the films after 6 days for PLA and PLA crystallized.

The degradation behavior of PLA films is observed in Figure 2. The samples become wither after one day due to a secondary crystallization phenomenon. It has been normally observed in the aging of plastic samples. Afterward, the degradation starts on the edges and film surface, probably due to the easy accessibility of the enzyme in these areas and continues to the center. After 6 days, the samples are almost disintegrated. Different behavior is observed for crystallized PLA. The initial sample is opaque, no transparent, due to the annealing performed during the cooling down of the processability to increase the crystallinity in the sample. In addition, a similar whiteness is kept constant on the sample while at the same time no holes and broken structures are observed in the films. Thus, it could indicate no degradation is observed in crystallized samples.

**Figure 3:**
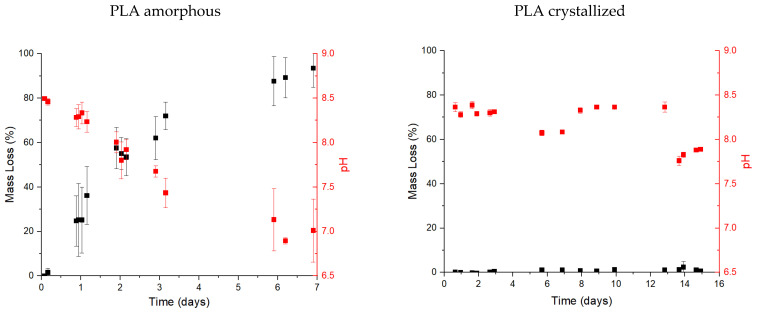
Biodegradation test of PLA (**left**) and PLA crystallized (**right**) in terms of mass loss (%) and pH.

Nonetheless, the optical analysis of the films seems to indicate a degree of degradation on the amorphous PLA samples and no effect in crystallized ones, but it needs to be confirmed. The results from the gravimetrical test and the changes reported in the pH are shown in Figure 3. As no degradation seemed to occur on the crystallized samples, the test was enlarged until 15 days.

Full disintegration of the films is obtained after 7 days in the buffer solution for the PLA samples. The stability of the films became quite irregular after one day when the sample obtained a significant degradation (over 20% of the sample mass). After 7 days it was impossible to measure the films samples as they were disintegrated. Small particles could be seen in the solution, but the weight was negligible. Thus, the oligomers produced from PLA degradation have to be smaller enough to be solved in the enzyme solution. The clear reduction in the pH is devoted to the chain scission of PLA into smaller chains. The scissions of the chain produced a higher number of acids groups at the end of the chains. The enzyme solution is prepared with a buffer solution, so the reduction of the pH to values equal or lower than 7 indicates a huge quantity of acid groups in the media to counteract the buffer effect. A blank test was performed without the presence of the enzyme but using the same buffer and films. No degradation was observed in these films while the pH was maintained at 8.6. Thus, all the degradation is devoted to the enzyme activity as at the used temperature, 37 °C; no hydrolysis is obtained.

In the case of the crystallized samples, the loss mass is really poor, with an average value of around 1% *w*/*w*. This limited degradation is related to the high degree of crystallization (44% initially calculated by DSC) in comparison with the obtained in the amorphous films (12%, also calculated by DSC). The presence of high crystalline domains inhibits the enzyme scission of the chains and its attachment to the material and diffuses through it. Nonetheless, it is interesting to comment on the reduction of the pH. The reduction trend is slower than in the case of the amorphous PLA where values lower than 7 are obtained. In this case, the lower values are around 7.8 after 14 days. However, the reduction of the pH is related to the apparition of acids groups in the media which could be due to the enzyme continuing the chain scission in the degraded part of the film. Thus, as it could not continue the degradation in the film, it could continue degrading the oligomers of the solution which come from the degraded part in the film.

**Figure 4:**
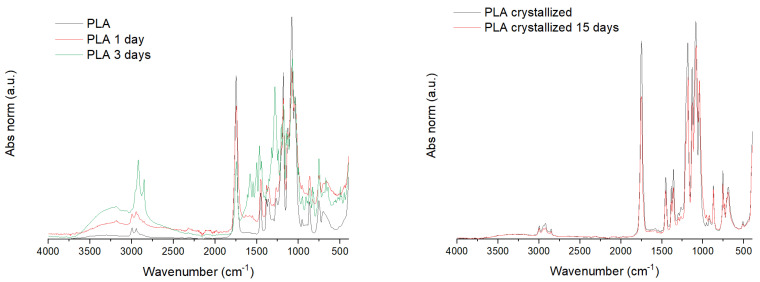
FT-IR of PLA amorphous and crystallized samples and some degraded specimens.

An FT-IR of the degraded samples was performed to analyze this enzyme behavior (Figure 4). The FT-IR absorbance values were normalized to the C–CH_3_ stretching peak as it is a band with medium intensity, common for all the PLA oligomers, including the monomer of PLA, lactic acid and it is poorly affected by the crystallinity degree and forms of PLA [55,56].

Few differences are observed between the PLA amorphous and crystallized. The most important and the most reference peak of crystallinity is the small band at 924 cm^−1^, characteristic of the helical conformation of PLA crystals. That band is appreciated in the PLA crystallized samples but not in the PLA amorphous initial and degraded samples. The degradation of the samples in the PLA amorphous films is proved in the FT-IR by the increment of the hydroxyl band. It is unappreciated in the starting material while increase with time. The increment of the intensity in such a band is due to the hydrolysis of the ester bonds, obtaining acid groups. Thus, it indicates that the chains in the film are getting hydrolyzed as considered previously. Conversely, the crystallized film does not suffer any difference during the 15 days of the test, in agreement with the observed in the gravimetrical test. Thus, the reduction in the pH of the medium has to be devoted to the hydrolysis of the small part degraded.

**Figure 5:**
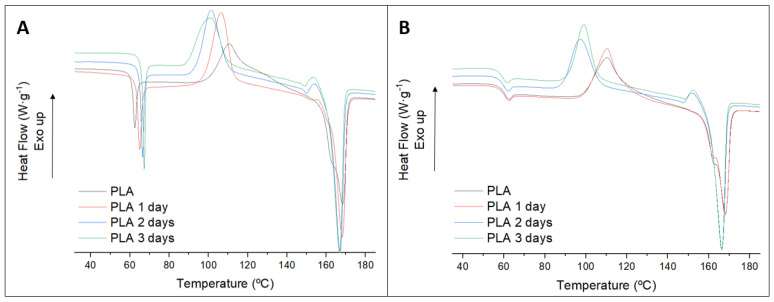
First (**A**) and second (**B**) heating of PLA samples from the degradation test.

DSC of the initial sample and degraded samples were also performed. Figure 5 shows the thermograph of the PLA and PLA degraded until the 3rd day of the test and during the first (A) and the second (B) heating.

The effect of the degradation is appreciated in the thermograph of the first heating. The aging and the increment of the crystallization are appreciated in the glass transition temperature (T_g_), which is displaced to higher temperatures. Additionally, the intensity and the cold crystallization temperature are affected: the cold crystallization temperature is shifted to lower temperatures and the peak increased, due to the smaller oligomers which required lower energy to be organized. The melting temperature is slightly affected in terms of average temperature, a small reduction of the melting temperature is observed, but the melting behavior changes. The initial sample of PLA showed a broad shoulder indicating probably different crystals sizes, but the degraded samples tend to a single peak. Moreover, the intensity of the melting process is increased due to the higher crystallinity of the samples. The initial crystallinity of the samples increased with the degradation time, from 12% of the initial sample to almost 20% after 3 days.

In the second heating, after erasing the thermal history, the effect of the degradation is observed with a lower impact. The cold crystallization and the melting temperatures are slightly lower than the initial ones, but the difference is smaller than observed in the 1st heating. The T_g_ showed differences also less than 1 °C. In addition, the crystallinity of the melting is increased, from 43% to 50% after 3 days, although the thermal history was erased. It is indicative of the degraded part being arranged easily.

**Figure 6:**
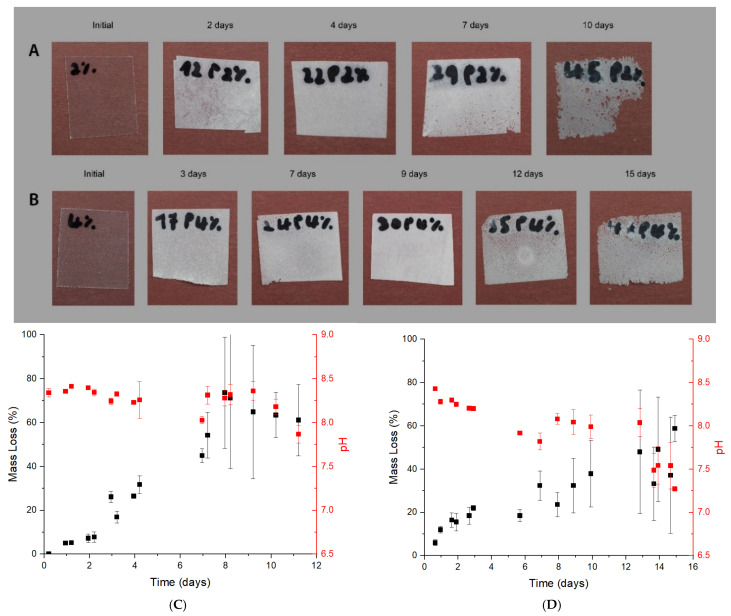
Optical analysis and gravimetrical test of nanocomposites reinforced with a 2% (**A**,**C**) and 4% (**B**,**D**) of nanoclays.

The effect of nanoclays on biodegradation was analyzed in the composites with 2 and 4% *w*/*w*. Figure 6 shows the optical analysis, gravimetrical results and the pH of the samples. Time was enlarged to 11 and 15 days for the 2 and the 4% *w*/*w*, respectively.

In both cases, the samples were not completely disintegrated at the studied times. Furthermore, the optical analysis showed a slow rate of biodegradation when the nanoclays contents are increased in the nanocomposites. The biodegradation pathway seems to be the same, starting from an increment of the crystallinity from the aging which turns white the sample and the mass loss in the edges, but the time has been enlarged. This effect is in agreement with the observed in the gravimetrical studies. Again, the samples were demonstrated to not be complete physically degraded. However, the error of the analysis became so big to ensure it, at least in the 2% reinforced nanocomposite which obtained values of full disintegration. These huge differences could be related to the nanoclays not totally homogeneously dispersed in the materials, leading to differences in the film samples and producing significant errors when the biodegradation rate in the samples is high. Moreover, the enzyme starts the degradation of the films in the surface as it is the most available part. The presence of these nanoclays in the surface could inhibit the enzyme biodegradation as the enzyme could be probably attached to the nanoclays surface. It is in agreement with that observed in the gravimetrical test. On the other hand, a different trend is appreciated after pH determination. In the 2% reinforced nanocomposite, the pH obtained is lower than the observed in the 4% at a similar loss mass. It seems that in the 2% the higher availability of the polymer could lead to oligomers production with higher molecular weight. The result is similar to the observed in PLA at similar degradation values. In the case of 4% *w*/*w* nanocomposite, the behavior is similar to the PLA crystallized as the pH of the samples reduced changed significantly although not similar values of mass loss are achieved. These results are in concordance with the hypothesis of the nanoclays inhibition of the film degradation. In addition, the biodegradation occurred and just the time is enlarged, which could indicate an enlargement of the lifespan of such films.

**Figure 7:**
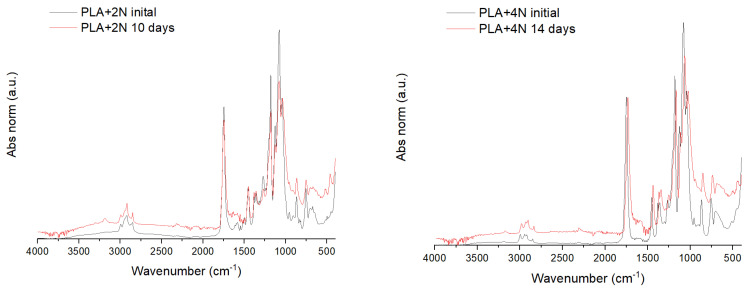
FT-IR of PLA reinforced with 2 (**left**) and 4% *w*/*w* (**right**) and its degraded samples after 10 and 14 days, respectively.

Structural changes in the film were analyzed by FT-IR and normalized using the same band. Figure 7 shows the obtained spectra for the 2 and 4% reinforced nanocomposites. A lower degradation of both materials is observed in comparison with PLA. The nanocomposite with 2% of nanoclays reported an average degradation of around 65 ± 11% of mass loss. Although the band of hydroxyl groups has appeared, it has not the same intensity as in PLA after 3 days, although the average mass loss is close. In the case of the nanoclay 4%, the band is still less appreciable. The result is expected as the oligomers which produce the reduction in the pH are probably soluble and do not remain in the film.

**Figure 8:**
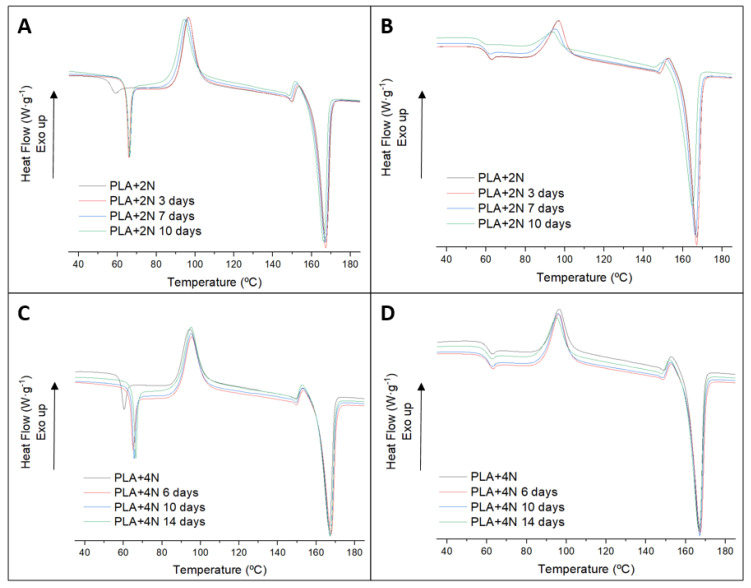
First, (left column) and second (right column) of nanocomposites: 2% (**A**,**B**) and 4% (**C**,**D**) of nanoclay content.

The thermal characterization of the nanocomposites and their degraded samples showed similar profiles in the first and the second melting (Figure 8). Samples and timings were chosen to have similar degradation rates to the ones previously analyzed in Figure 5 for PLA. A summary is included in the supporting information (Table S1). The first heating of both nanocomposites showed the effect of the aging in a marked Tg. In addition, in between the degraded samples, the Tg remains similar. In addition to this result, the other main transitions are similar, except for the cold crystallization of the second melting of PLA + 2%NC. In that case, the temperature becomes lower for the more degraded samples. These results are related to the higher molecular weight of the PLA chains in the nanocomposites, predicted in the behavior of the gravimetrical test.

### 3.3. Processability

An important aspect is the thickness of the film. Films of 100–250 μm were used for this experiment. A thickness of 100 μm is usual in the food packaging industry. It is, therefore, important to gain more information about the processability of these polymers with this thickness. This determines the viability of these polymers as easy manufacturable, competitive food packaging possibilities.

Once the processing conditions were set up, all the specimens showed a good ability to be transformed by classical thermoforming. The mold was male; thus, a 1:1 ratio between the base aperture and height was applied [57]. The film bore the first stage of the process and no tears appeared during its mechanical stretching. When the vacuum was applied, all the films adhered to the mold finishing the process. There were no wrinkles, nor an orange peel effect. The molding condition was stored and used for the next phase. The inclusion of nanoclay did not affect the thermoformability of the films. Furthermore, the time needed to reach thermoform temperature was reduced a 50%, with the involved energy savings.

To pre-evaluate the ability of the films to wear an extrusion blown molding process that involves higher deformations than a thermoforming, the films were stretched with a prismatic solid. A set of films were tested and it was found that at certain deformation heights tears appeared on the surface of the films. The maximum deformation was obtained for a PLA film and a 152 mm height. On the other hand, it was observed that for deformation below 116 mm, some materials did not produce tears on their surface. This height was proposed as a control. The prismatic solid was mechanized up to this height and a new series of high height thermoforming was started. The films submitted to these experiments hold the node aided stretching phase but did not adhere to the mold when the vacuum was applied due to the presence of tears in the film surface or the temperature of the film. Regardless, this last phase was not required to evaluate the ability of the films to endure high deformations.

**Figure 9:**
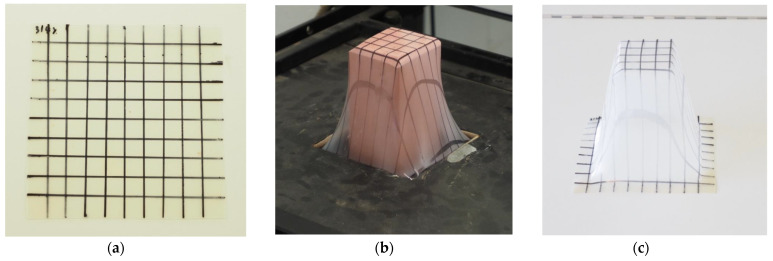
Different stages thermoforming, (**a**): film with grid pattern; (**b**): the film being thermoformed; (**c**): the thermoformed film.

Figure 9 shows different stages of high height stretching experiment.

Parallel lines at 10 mm intervals in the x and y directions were drawn in the film surfaces to evaluate the zones submitted to higher deformation (Figure 9a). Figure 9b shows the parallelepiped node used to assist the deformation of the films. The figure shows the node at its maximum height. Figure 9c shows the thermoformed film extracted from the equipment.

Four parameters were examined to characterize the processability of the films: the maximum height of node assist formability, the first height at which a tear appears in the thermoformed film, the aerial draw ratio (ADR) and the film wall thickness reduction (WTR).

Table 2 shows the maximum height of node assist formability (HNAF) and the distance to the first tear (DFT).

Table 2 demonstrates that PLA has the highest thermoformability of the three types of polymers with a thermoformed height of 152 mm. PLA with 2 *w*/*w*% and 4 *w*/*w*% nanoclays both have similar thermoforming height, even though there is a difference in nanoclay mass percentage. These results could explain that the introduction of nanoclays causes the polymer matrix to be less able to deform into larger shapes. It could be that nanoclay content gives irregularities inside of the polymer, causing stress concentration phenomena.

It was noticed that composites were less likely to have a tear than the matrix. No tears were found for PLA 2% and PLA 4% when the thermoforming height was 116 mm. This can open the use of such materials for film blown molding, but more experimentation is needed. Being better and more efficient processing in an industrial setting, with better control of parameters such as temperature and speed of thermoforming, could give better thermoforming. The ability to use film blown molding with the PLA composites will be further discussed with ADR results.

The aerial draw ratio (ADR) is used to characterize the processability of the polymer. It is an instructional tool for comparing part designs and processes. ADR can be described numerically when the surface area can be calculated. ADR is defined by the ratio between the area of the thermoformed film and the area of the original film. As soon as the increase of area is directly proportional to the film thickness reduction, the percentage thickness reduction (PTR) can be obtained as 1-1/ADR (%).

**Figure 10:**
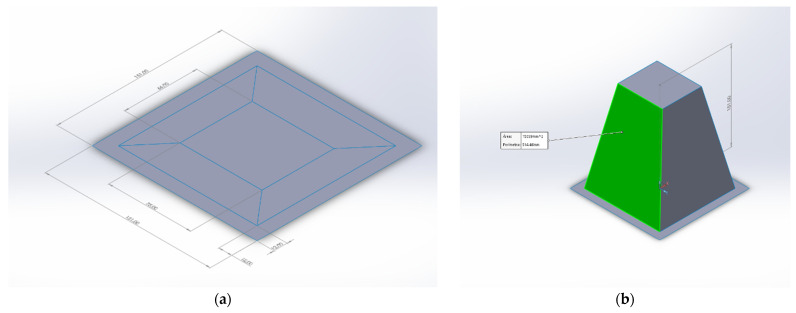
Model used to compute an approximation to the area of the thermoformed film: (**a**) Original film and area definition (**b**) Thermoformed model.

The surface area of the thermoformed part is difficult to by measured and is calculated from a simplified geometry (Figure 10).

The plastic film model is designed with the same dimensions and shape as the real thermoformed film (Figure 10a). Three zones are defined in such a planar area. The outer area is attached to the thermoforming equipment frame and does not deform. This area measures 152^2^ − 128^2^ mm^2^ = 6720 mm^2^. This area is not submitted to any deformation and is excluded from ADR calculations. Thus, the area of the original film will be considered as 128^2^ mm^2^ = 16384 mm^2^. The inner zone coincides with the top of the node used to deform the film and measures 66·75 mm^2^ = 4950 mm^2^. The area of these zones remains unchanged after thermoforming. The zone between the inner and the outer area transforms from plane to the sides of a truncated pyramid (Figure 10b). The area of the thermoformed film against thermoforming height (H) can be obtained from:

(4) $$A=4950+2\times \left(\frac{127+66}{2}\times \sqrt{H^{2}+26^{2}}\right)+2\times \left(\frac{127+75}{2}\times \sqrt{H^{2}+30.5^{2}}\right)$$

Table 3 shows the computed ADR and PTR for the different films and experiment data.

The results show how PLA was able to withstand the highest deformations but this was accompanied by tear creation. Thus, although materials were able to deform up to considerable amounts, only materials that showed no tears are interesting for film blow molding. In this sense, PLA 25 and PLA 4% at 116 mm meet this condition. This means an ADR of 3.63 and a theoretical average thickness reduction of 68.9%.

Blow-up ratio (BUR) is the ratio between the bubble diameter and the die diameter in blown extrusion equipment. The increase in the diameter of the bubble is linked to the thickness decrease of the film. Using data in Table 3 and a die diameter of 55 mm [49], the corresponding bubble diameter can be up to 55·ADR = 339 mm. This value is inferior to the 410 mm obtained with PLA and poly(butyleneadipate-co-terephthalate) (PBAT) and poly(butylene succinate) (PBS) in the presence of polypropyleneglycol di glycidyl ether (EJ400) [49] (but is not far). It must be considered that extrusion blown molding uses pressure to obtain the deformation and is applied to an extruded section at a constant temperature. Thermoforming is less a continuous process and more prone to be influenced by defects in the surface of the film.

**Figure 11:**
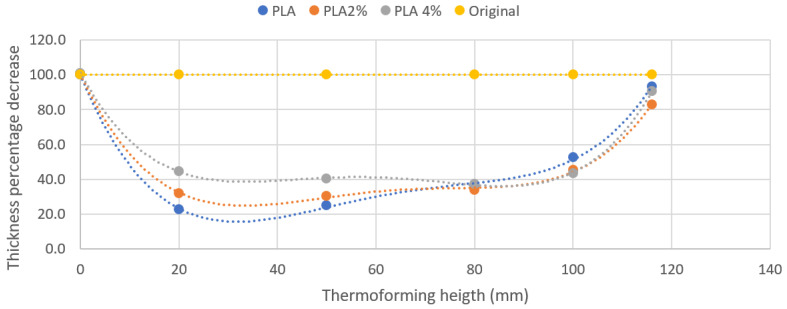
Experimental thickness of the central line of a thermoformed film against thermoforming height.

Regarding thickness reduction, in extrusion blow molding equipment, such decrease is constant. In the case of thermoforming, thickness reduction changes with the height and with the composite formulation. Figure 11 shows a profile of the central line of a thermoformed specimen.

In the figure, it can be seen how the main thickening occurs in the vicinity of the base where the thickness of the film fatly decreases up to 20% of the initial value for PLA. In the top region, the thickness of the film was also lower than the initial due to creep from the upper area to the side areas. The presence of nanoclays favored a more stable thickness reduction for the lateral sides. Nanoclays acted as plasticizers allowing a more regular flow of the material.

## 4. Conclusions

The effect of the nanoclays content in the PLA matrix was analyzed in terms of biodegradability and processability. Firstly, PLA nanocomposties were tested in WVTR and global migration to demonstrate their suitability for food contact applications. The WVTR was reduced by the addition of the nanoclays while the global migration was under 1 mg per kg and dm^2^; the limit was 10 mg. The biodegradation of the nanocomposites was enlarged due to the presence of nanoclays. In the case of PLA, a maximum of 7 days was required while for a 4% *w*/*w* content it was increased up to more than 15 days. In addition, the enzyme was able to degrade the nanocomposites and the time enlargement could allow increasing the lifespan of the films. Regarding the processability, the use of nanoclay as PLA fillers showed a higher ability than the matrix to sustain elongation without tear creation. This increased the ability of such composites to be processed under thermoforming or even film blow molding, increasing the percentage of nanoclay produced thermoformed films with lower wall thickness oscillations, showing that nanoclay acted as plasticizing agents.

## Figures and Tables

**Table 1:** Global migration results for PLA and PLA+4%NC in water, used as reference, and simulant B.

Sample	Water		Simulant B	
	Migration (mg/kg)	Migration (mg/dm^2^)	Migration (mg/kg)	Migration (mg/dm^2^)
PLA	<1	<1	<1	<1
PLA + 4%NC	<1	<1	<1	<1

**Table 2:** Maximum heights obtained by node assist formability before film breakage and distance from the top formed specimen to the first tear. Different letters ^a, b, c^ represent the statistical difference (ANOVA, *p* < 0.05) between the properties of the materials.

Material	PLA	PLA	PLA 2%	PLA 2%	PLA 4%	PLA 4%
HNAF (mm)	116 ± 2 ^a^	152 ± 1 ^b^	116 ± 2 ^a^	135 ± 8 ^c^	116 ± 4 ^a^	137 ± 4 ^c^
DFT (mm)	17.6 ± 5	10.7 ± 6	0	35.1 ± 6	0	45.1 ± 7

**Table 3:** Area of thermoformed shapes (A), aerial draw ratio (ADR) and percentage thickness reduction (PTR) for the specimens transformed using different PLA-based materials and different heights.

Material	PLA	PLA	PLA 2%	PLA 2%	PLA 4%	PLA 4%
HNAF (mm)	116	152	116	135	116	137
A (mm^2^)	52,121	66,028	52,121	59,441	52,121	60,214
ADR	3.18	4.03	6.18	3.63	3.18	3.68
PTR (%)	68.6%	75.2%	68.6%	72.4%	68.9%	72.8%

## Supplementary Materials

The following are available online at https://www.mdpi.com/article/10.3390/polym13162741/s1, Table S1: DSC samples indicating time and average mass loss (%).
