# Supplementary material for: Nanoclay Effect into the Biodegradation and Processability of Poly(lactic acid) Nanocomposites for Food Packaging
Source: Polymers (Basel). 2021 Aug 16;13(16):2741. doi: 10.3390/polym13162741 (PMC8399732; doi:10.3390/polym13162741)
Supplement: Supplementary file 1 [file polymers-13-02741-s001.zip › polymers-1339678-supplementary.pdf]

Table S1. DSC samples indicating time and average mass loss (%).

Sample	Time (days)	Mass Loss (%)
PLA	0	0
	1	25
	2	55
	3	72
PLA+2N	0	0
	3	26
	7	45
	10	63
PLA+4N	0	0
	6	19
	10	38
	14	50
